# An unusual combined thymic carcinoma composed of squamous cell carcinoma and type AB thymoma: a rare case report

**DOI:** 10.1186/s13000-016-0590-3

**Published:** 2017-01-17

**Authors:** Yufeng Jiang, Yang Liu, Xiuying Shi, Xiaoyun Mao, Yang Zhao, Chuifeng Fan

**Affiliations:** 1 Department of Pathology, First Affiliated Hospital and College of Basic Medical Sciences of China Medical University, 110001 Shenyang, China; 2 97K Seven-year Program of Medicine, China Medical University, 110001 Shenyang, China; 3 Department of Breast Surgery, Department of Surgical Oncology, Research Unit of General Surgery, the First Affiliated Hospital of China Medical University, 110001 Shenyang, China; 4 Department of Hepatobiliary and Spleenary Surgery, The Affiliated Shengjing Hospital, China Medical University, 110004 Shenyang, China

**Keywords:** Combined thymic carcinoma, Mediastinum, Squamous cell carcinoma, Type AB thymoma

## Abstract

**Background:**

Combined thymic carcinoma is a malignant neoplasm of the thymus recently added to the 4th edition of the World Health Organization (WHO) classification of tumors of the lung, pleura, thymus and heart. It involves at least one type of thymic carcinoma and another thymic epithelial tumor. The previously used term “combined thymic epithelial tumor” has been abandoned.

**Case presentation:**

Here, we present an unusual case of combined thymic carcinoma of the thymus in a 44-year-old male who had suffered from fever, chest pain, chest tightness and shortness of breath. Magnetic resonance imaging (MRI) detected a mass approximately 6.4 cm × 4.2 cm in the anterior mediastinum, and a nonencapsulated tumor approximately 5.0 cm × 3.5 cm × 2.5 cm with an irregular shape was resected. The morphological features and the immunostaining pattern of the tumor revealed it to be an unusual combined thymic carcinoma consisting of type AB thymoma and squamous cell carcinoma. There were cysts of various sizes, some of which had crack-like structures, in the type AB thymoma area. A gradual transition could be seen between these structures and the squamous cell carcinoma, indicating that the carcinoma portion may have originated from the composition of the thymoma.

**Conclusions:**

Combined thymic carcinoma composed of type AB thymoma and squamous cell carcinoma is rare, and the carcinoma portion may have originated from epithelial structures in the type AB thymoma.

## Background

Combined thymic carcinomas are tumors composed of at least one type of thymic carcinoma and another thymic epithelial tumor of any type of thymoma or thymic carcinoma, excluding small cell carcinoma and large cell neuroendocrine carcinoma [1]. It was proposed that the previously recommended term “combined thymoma” be abandoned because thymomas of different histological types within the same tumor are very common, according to the 4th edition of the World Health Organization (WHO) classification of tumors of the lung, pleura, thymus and heart [1]. Instead, the WHO suggested that all the histological types present be listed in the diagnosis [1]. For heterogeneous tumors that consist of a thymic carcinoma and a thymoma, the organization proposes the new term “combined thymic carcinoma.” The most common combinations are thymic squamous cell carcinoma and type B3 thymoma and papillary adenocarcinoma or sarcomatoid carcinoma associated with type A thymoma [1–4]. The different components of the tumors may show a gradual transition or be sharply separated from each other [1]. Thymic carcinoma occasionally arises in type A thymoma, but combined thymic carcinoma composed of squamous cell carcinoma and type AB thymoma is extremely rare. Here, we present a case of combined thymic carcinoma composed of type AB thymoma and squamous cell carcinoma using the diagnostic criteria of the 4th edition of the WHO classification of tumors of the lung, pleura, thymus and heart.

## Case presentation

### Clinical history

A 44-year-old man was referred to our hospital with a 1-month history of fever without an obvious cause. In that period, after he had taken anti-inflammatory agents the fever subsided to some extent. However, in the week before presenting to the hospital for diagnosis and treatment, he experienced chest pain, chest tightness, shortness of breath, severe cough and blood in phlegm accompanied by vomiting after tiredness. In examination at the hospital, he reported no muscle weakness. Blood tests showed neuron-specific enolase (NSE) (19.71 ng/ml), CA125 (49.61 μ/ml) and Cyfra21-1 (8.42 ng/ml) levels slightly higher than normal, whereas his CEA and CA199 levels were normal. The patient underwent surgery, and the tumor was found to have invaded the local pericardium. No lymph node metastasis or distant metastasis was detected. The patient showed no recurrence 6 months after the surgery.

## Materials and methods

The resected samples of the tumor were embedded in paraffin blocks. The blocks were cut into sections and stained with alum hematoxylin and eosin for morphological examination under light microscope. The immunohistochemistry was examined using an SP-kit (Maixin Biotechnology, Fuzhou, Fujian, China) according to the instruction manual of the developer. The sections were incubated overnight at 4 °C with the following primary antibodies: CD1α (1:100, DAKO), CD3 (1:100, DAKO), CD5 (1:200, DAKO), CD20 (1:100, DAKO), CD99 (1:200, DAKO), CK (Pan) (1:200, DAKO), CK19 (1:200, DAKO), CK20 (1:100, DAKO), Ki-67 (1:200, DAKO), p63 (1:100, DAKO) and TdT (1:100, DAKO). This study was prospectively performed and approved by the institutional ethics committees of China Medical University and conducted in accordance with the ethical guidelines of the Declaration of Helsinki.

## Results

### Imaging and gross features

**Fig. 1:**
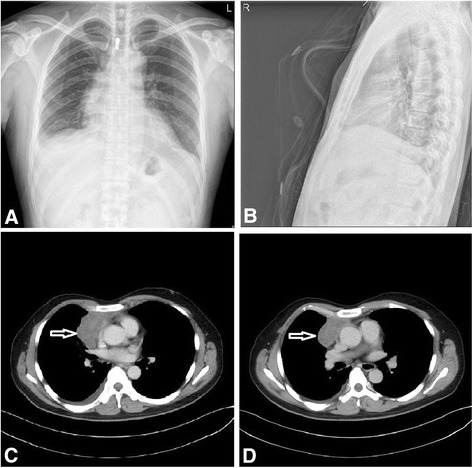
The imaging of the tumor. The X-ray shows a widened mediastinum without displacement (**a**, **b**). MRI detected a mass that was approximately 6.4 cm × 4.2 cm in the anterior mediastinum beside the right part of the heart (**c**, **d**). The margins between the mass and the pericardium were not clear

Figure 1 shows the X-ray and MRI findings. The X-ray showed a widened mediastinum without displacement (A, B). The lung field was clear and the size of the heart was normal. MRI detected a mass approximately 6.4 cm × 4.2 cm in the anterior mediastinum beside the right part of the heart (C, D). The margins between the mass and the pericardium were not clear. The surgical records indicated that the tumor was located on the right side of the anterior mediastinal and was closely attached to part of the right lung, the superior vena cava and the ascending aorta. The resected tumor was approximately 5.0 cm × 3.5 cm × 2.5 cm. It was nonencapsulated with an irregular shape. The cut surface of the mass was gray and firm. Hemorrhage, necrosis and cyst formation were observed in some areas.

### Microscopic features

**Fig. 2:**
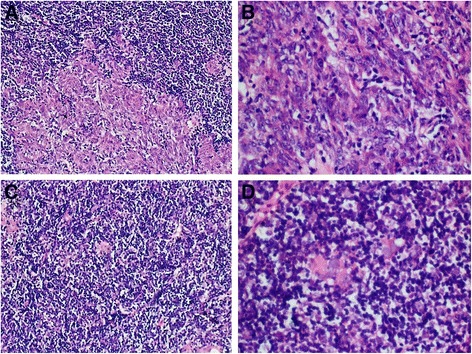
Morphological features of the tumor. Part of the tumor was composed of AB thymoma (**a**). The type A region of the thymoma consisted mainly of oval tumor cells arranged in nests with a few dispersed lymphocytes (**b**). The tumor cells had bland nuclei, dispersed chromatin and inconspicuous nucleoli (**b**). The type B area consisted mainly of lymphocytes with few small polygonal epithelial cells with bland nuclei (**c**, **d**)

**Fig. 3:**
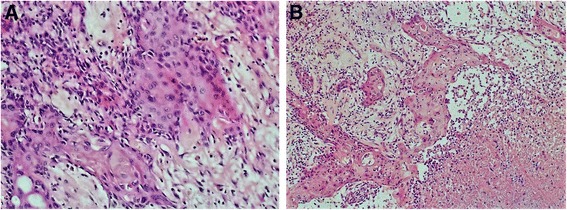
Morphological features of the tumor. Part of the tumor was composed of keratinizing squamous cell carcinoma (**a**,**b**) with necrosis and hemorrhage (**b**)

**Fig. 4:**
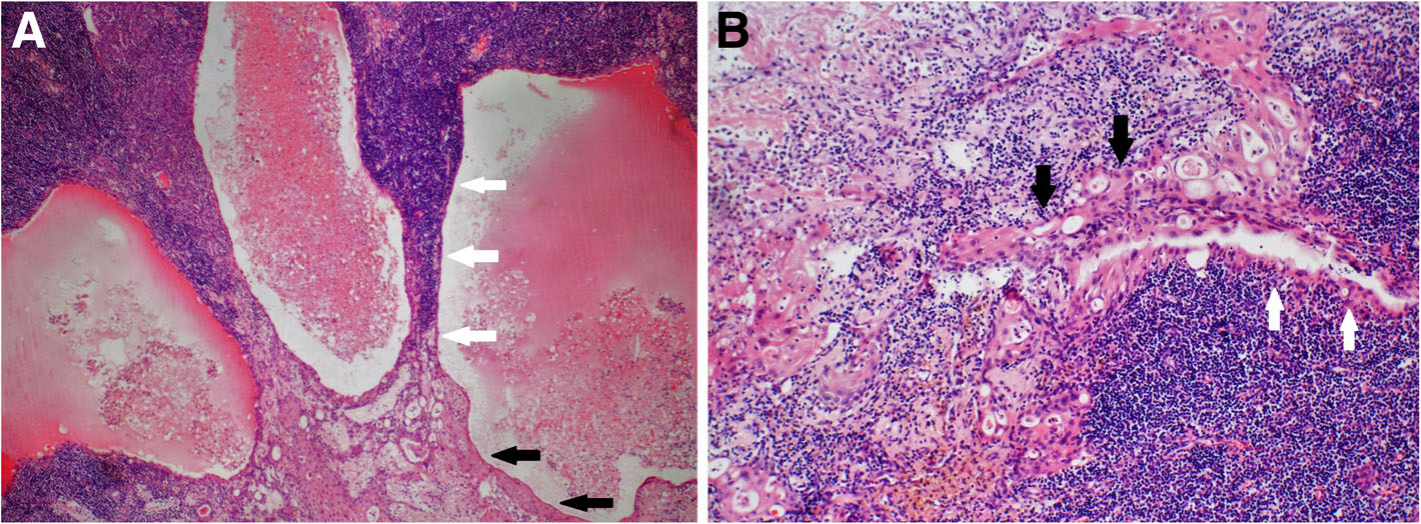
Morphological features of the tumor. There were cysts of various sizes, some of which had crack-like structures, in the area of the AB thymoma (**a**, **b**). A gradual transition was found between these structures in the thymoma and squamous cell carcinoma. The white arrows indicate the epithelial cells of the cystic structures in the thymic thymoma and the black arrows indicate the carcinoma cells of the squamous cell carcinoma

Figure 2 shows that thymoma accounted for approximately 50% of the tumor tissue. Panel A shows that the histological type of the thymoma was AB thymoma. Panel B shows that the type A region of the thymoma consisted mainly of oval tumor cells arranged in nests with a few dispersed lymphocytes. The tumor cells had bland nuclei, dispersed chromatin and inconspicuous nucleoli. Panels C and D show that the type B area consisted mainly of lymphocytes with a few small polygonal epithelial cells with bland nuclei. As shown in Fig. 3, another part of the tumor was keratinizing squamous cell carcinoma, accounting for approximately 50% of the tumor tissue (A, B). There was marked necrosis and hemorrhage in this region of the tumor (B). Fig. 4 shows that in the area of the AB thymoma, there were cysts of various sizes, some of which had crack-like structures (A, B). There was a gradual transition between these structures in the thymoma and squamous cell carcinoma (A, B), which indicates that the squamous cell carcinoma may have originated in the thymoma rather than being a metastatic tumor.

### Immunophenotype

**Fig. 5:**
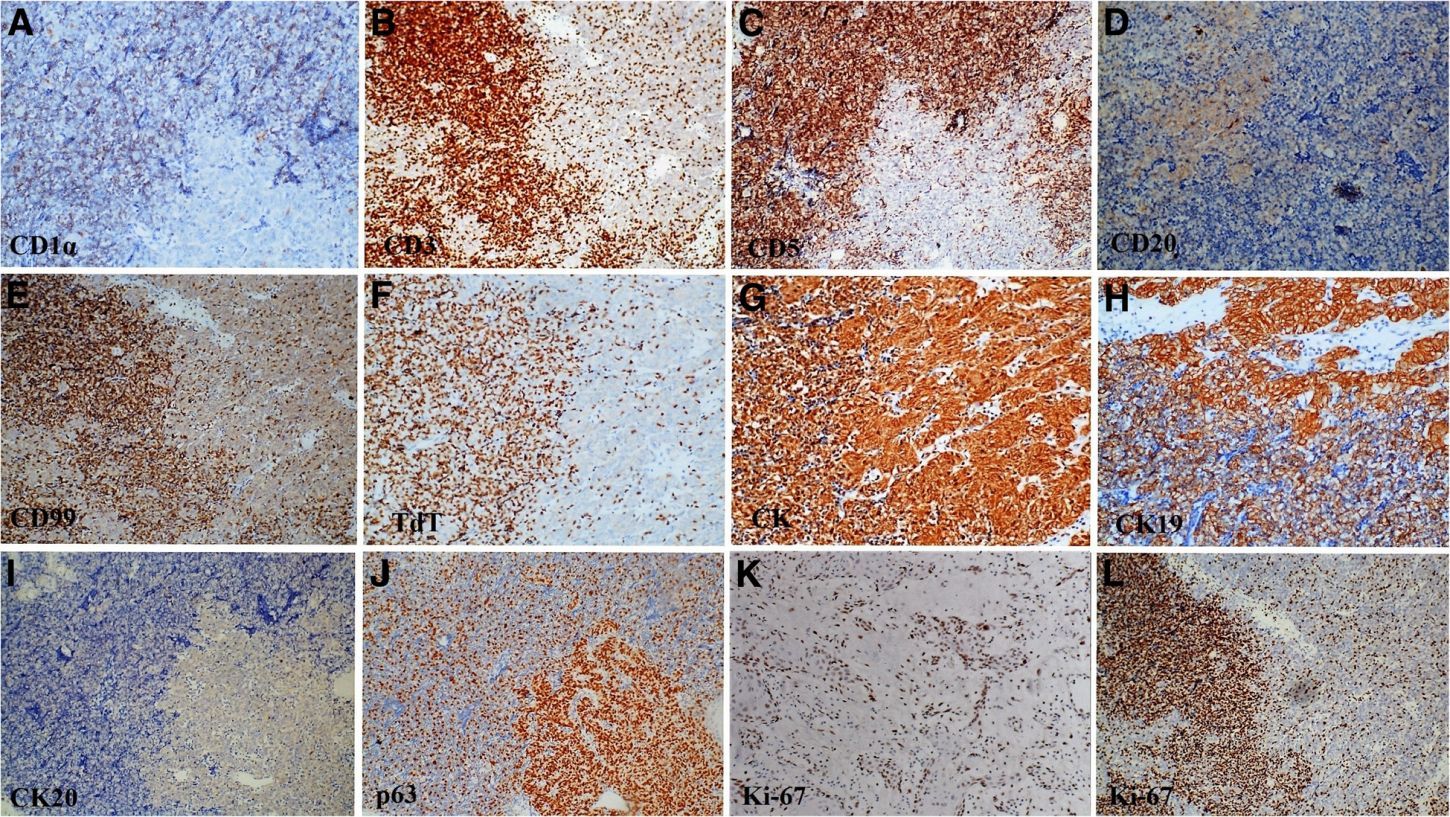
Immunostaining pattern of the tumor. CD1α was positive mainly in the cytoplasm of the T lymphocyte cells in the type B area in type AB thymoma (**a**). The immunostaining patterns of CD3, CD5, CD99 and TdT were similar to that of CD1α, which indicates immature T lymphocyte cells in these areas (**b**, **c**, **e**, **f**). There were very few CD20-positive cells in the tumor tissue (**d**). CK and CK19 were positive in the cytoplasm of the epithelial cells in both the type A and B areas of type AB thymoma, but the immunostaining was stronger and more diffuse in the type A area than in the type B area (**g**, **h**). CK20 was negative (**i**). P63 was positive mainly in the nucleus of the epithelial cells in both type AB thymoma and squamous cell carcinoma (**j**). The Ki67 index in squamous cell carcinoma was approximately 50% (**k**). It was nearly 100% in the lymphocytes in the type B area in type AB thymoma but was very low at (approximately 3%) in the epithelial cells in the type A area (**l**)

**Fig. 6:**
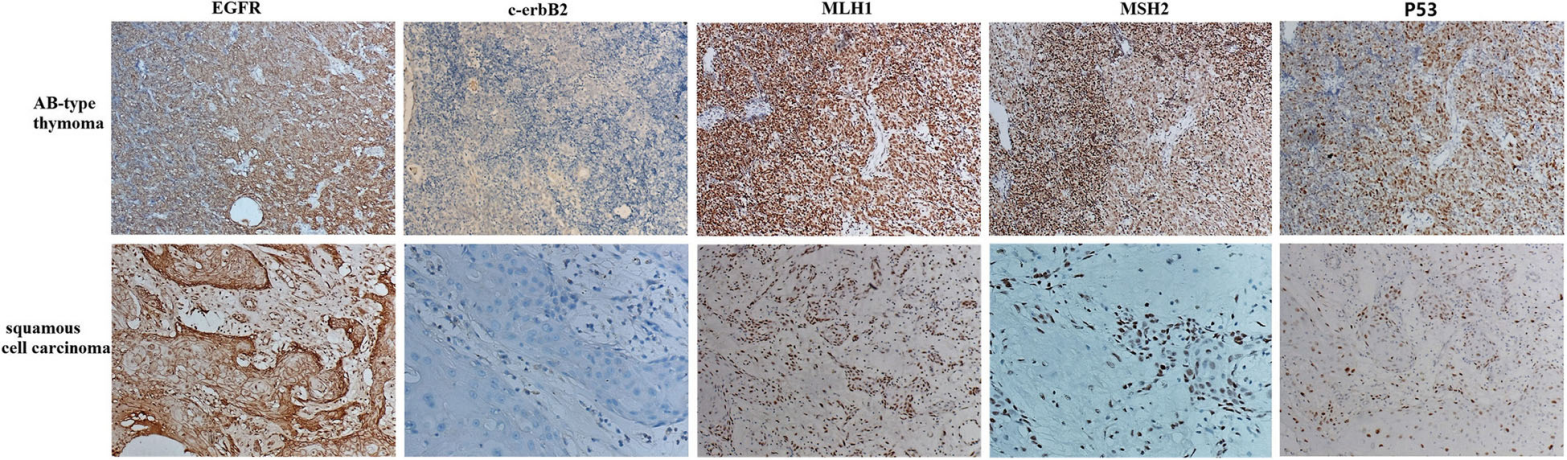
Genetic profile of the tumor via immunohistochemistry. The immunostaining patterns of EGFR, c-erbB-2 and P53 in type AB thymoma and thymic carcinoma were similar, but there was greater MLH1 and MSH2 loss in thymic carcinoma (>10%) than in type AB thymoma (<10%)

Figure 5 shows the immunostaining pattern of the tumor. CD1α was positive mainly in the type B area in type AB thymoma (Fig. 5a). Cells positive for CD3 (Fig. 5b) and CD5 (Fig. 5c) were also seen mainly in the type B area in type AB thymoma. There were very few CD20-positive cells in the tumor tissue (Fig. 5d). The pattern of CD99 (Fig. 5e) and TdT (Fig. 5f) immunostaining was similar to that of CD1α, CD3 and CD5 in type AB thymoma and squamous cell carcinoma. These results indicate that the immature T lymphocytes were found mainly in the type B area in type AB thymoma. CK (Fig. 5g) and CK19 (Fig. 5h) were positive both in the type A and B areas of type AB thymoma, but the immunostaining was stronger and more diffuse in the type A area than in the type B area. CK20 was negative (Fig. 5i). p63 was positive in both type AB thymoma and squamous cell carcinoma (Fig. 5j). CD5 and CD117 were negative in squamous cell carcinoma. The Ki67 index in squamous cell carcinoma was approximately 50% (Fig. 5k). It was nearly 100% in lymphocytes in the type B area in type AB thymoma but was very low (approximately 3%) in epithelial cells in the type A area (Fig. 5l). We also examined the genetic profile of the tumor via immunohistochemistry. Figure 6 shows that the immunostaining patterns of EGFR, c-erbB-2 and p53 in type AB thymoma and thymic carcinoma were similar, but there was greater MLH1 and MSH2 loss in thymic carcinoma (>10%) than in type AB thymoma (<10%), which indicates the presence of microsatellite instability in thymic carcinoma but not type AB thymoma.

## Discussion

Primary thymic carcinomas are rare malignant neoplasms of the mediastinum, and combined thymic carcinomas are even less common [1–4]. Almost all reported cases of combined thymic carcinomas were located in the anterior mediastinum [1]. The clinical features of the tumors are the same as those of thymic epithelial tumors of a single histological type [1, 5, 6]. Most components of carcinoma in combined thymic carcinomas are squamous cell carcinomas, and type B2 and B3 thymomas account for most of these components [1–4]. Thymic carcinoma combined with type A thymoma is rare, but combined thymic carcinoma composed of squamous cell carcinoma and type AB thymoma is even less common. The macroscopic features of combined thymic carcinoma are the same as those of tumors of a single histological type [1–4]. The case reported here was diagnosed as an unusual combined thymic carcinoma composed of type AB thymoma and squamous cell carcinoma according to the 4th edition of the WHO classification of tumors of the lung, pleura, thymus and heart. This type of thymic tumor is new to this edition, and it is suggested that the formerly used term “combined thymic epithelial tumor” not be used anymore because thymomas showing different histological types in the same tumor are very common.

In the current case, histological and immunohistochemical findings revealed a combination of type AB thymoma and squamous cell carcinoma. There were cysts of various sizes, some of which had crack-like structures in the type AB area of thymoma, with a gradual transition between these structures and the squamous cell carcinoma. This indicates that the squamous cell carcinoma may have originated from the composition of the thymoma tissue. However, whether the carcinoma components originated from the epithelial cells in the thymomas and what mechanisms are involved remain unclear. Here, we found that CD5 and CD117 were negative in thymic carcinoma. Metastatic carcinoma needed to be ruled out. According to the 4th edition of the WHO classification of tumors of the lung, pleura, thymus and heart, the positive rates of CD5 and CD117 in thymic squamous cell carcinoma are 74% and 84%, respectively [1]. The physical examination of the patient did not indicate the presence of tumor in other part of the body other than the anterior mediastinum. The pathological findings indicated a transition between the squamous cell carcinoma and the cysts in the type AB thymoma. These findings suggest that the squamous cell carcinoma was originally part of a combined thymic tumor rather than a metastatic tumor. Thymic carcinoma can arise from thymic cysts. In this case, we found numerous structures of cysts in the portion of the type AB thymoma in the tumor tissue. There was a transition between this section and the squamous cell carcinoma. It indicated that there was a close relationship between these different tumor parts and that the portion of carcinoma may have originated in the thymoma. However, it does not entirely exclude the possibility that the carcinoma may have originated from a thymic cyst because the cyst might also have come from the structures of the cysts in the thymoma. Actually, in this case, the squamous cell carcinoma showed cystic change and hemorrhage, although we did not find a clear lesion involving a thymic cyst. Interestingly, CD5 and CD117 were both negative in the squamous cell carcinoma, which is often positive for these two markers. Whether there are different features of these two markers in the squamous cell carcinoma that originated from a thymic cyst compared with that in thymic squamous carcinoma needs to be further clarified. Moreover, the origination and oncogenesis of thymic squamous carcinoma are not fully understood. No systematic study has been done on the genetic changes in the components of combined thymic carcinoma to provide clues and evidence to answer these questions. Some studies have shown that the genetic alterations found in thymic squamous carcinomas of the thymus are distinctive from those in tumors of the lung, head, and neck, although their morphological features are similar [7–9].

## Conclusion

Combined thymic carcinomas are epithelial thymic tumors that consist of at least one type of thymic carcinoma and another thymic epithelial tumor. Although most of these tumors are composed of squamous cell carcinoma with type B2 or B3 thymomas, our study indicates that type AB thymoma can also be combined with squamous cell carcinoma in the anterior mediastinum. The gradual transition of the cystic structures in type AB thymoma with squamous cell carcinoma points to the possible origin of the malignant part of the thymoma, although systematic genetic analysis is needed to understand the etiology and carcinogenesis of these tumors.

## Abbreviations

MRI: Magnetic resonance imaging
WHO: World Health Organization
